# Retrospective analysis of jaw biopsies in young adults. A study of 1599 cases in Southern Brazil

**DOI:** 10.4317/medoral.21918

**Published:** 2017-10-21

**Authors:** Karine Silva, Alessandro Alves, Marcos Correa, Adriana Etges, Ana-Carolina Vasconcelos, Ana-Paula Gomes, Sandra Tarquinio

**Affiliations:** 1 DDS, MSc, Post Graduate Program in Dentistry, School of Dentistry, Federal University of Pelotas, Pelotas-RS, Brazil. Center of Diagnosis of Oral Diseases, School of Dentistry, Federal University of Pelotas, Pelotas-RS, Brazil; 2 DDS, MSc, PhD. Post Graduate Program in Dentistry, School of Dentistry, Federal University of Pelotas, Pelotas-RS, Brazil Unidade Integrada Vale do Taquari de Ensino Superior (UNIVATES), School of Dentristry, Lajeado-RS, Brazil; 3 DDS, MSc, PhD. Post Graduate Program in Dentistry, School of Dentistry, Federal University of Pelotas, Pelotas-RS, Brazil; 4 DDS, MSc, PhD. Post Graduate Program in Dentistry, School of Dentistry, Federal University of Pelotas, Pelotas-RS, Brazil Center of Diagnosis of Oral Diseases, School of Dentistry, Federal University of Pelotas, Pelotas-RS, Brazil

## Abstract

**Background:**

To evaluate the prevalence and the characteristics of jaw lesions diagnosed in young adults aged 20 to 30 years in a southern Brazil reference center, over a period of 25 years. And to analyze the concordance between clinical and histological diagnosis.

**Material and Methods:**

In this cross-sectional retrospective study, the biopsies files from this center were retrieved and data regarding sex, age, bone localization, clinical and histological diagnosis were collected. The histological diagnosis were grouped into the categories Cystic lesions of odontogenic origin, Periapical inflammation, Odontogenic tumors, Bone diseases, Health tissue and Nonspecific diagnostic. Absolute and relative frequencies were estimated with descriptive analysis. The agreement between clinical and histological diagnosis was measured through Kappa statistic.

**Results:**

A total of 18,181 histopathological analysis were performed during the period of the study, registering 1,599 jaw lesions in young adults. The average age of individuals was 24,59 years (SD 3,1). Nine hundred ninety-one (62%) lesions were found in females and 608 (38%) in males. More than half of pathologies were cystic lesions of odontogenic origin (822/51.4%), followed by periapical inflammation (282/17.6%). Regarding the site of lesions, more than half occurred in posterior mandible (877/54.8%), followed by posterior maxilla (339/21.2%). The most frequent entities were periapical cyst, chronic periapical granuloma, dental follicle and paradental cyst, corresponding to a total of 1,202 (75.2%) evaluated cases. In relation to the analysis of concordance between clinical and histological diagnosis the general Kappa index was 0.5, which is considered moderate. Finally, the findings confirm data from literature about the most frequent jaw pathologies in young adults and serve as aid for preventive measures of some entities. Additionally, they can improve the formulation of differential diagnosis and the patient management.

** Key words:** Jaw, biopsy, retrospective studies, young adult, Brazil.

## Introduction

Some aspects make the jaws different from other skeletal bones, such as their embryological development and because they anchor teeth. Therefore, diseases not found in other sites, such as odontogenic cysts and tumors may be present in the jaws (1). Periapical cyst and granuloma, dentigerous cyst, paradental cyst, odontoma, ameloblastoma and odontogenic keratocyst have been commonly reported in the literature, not only as case reports manuscripts, but as epidemiological studies (2-4).

While some of these pathologies are easily diagnosed, several of them have similar clinical and radiographic presentation, increasing the differential diagnosis list and greatly justifying histopathology evaluation, the standard diagnostic reference (5,6).

In this context, epidemiological studies are important in stomatology, since they provide professional help in the formulation of clinical hypothesis and offer data about the prevalence and characteristics of the most frequent pathological alterations in the study population (3,7,8).

It is known that young adults look for dental services more often, not only due to caries and periodontal diseases, but also for aesthetic and occlusal issues (9-12), making the determination of the prevalence of oral lesions in this age group very important. In addition, to the best of our knowledge, there are no similar studies conducted in any other regions of Brazil.

This study aims to evaluate the prevalence and characteristics of jaw lesions diagnosed in young adults aged 20 to 30 years in a southern Brazil reference center, over a period of 25 years.

## Material and Methods

The project was approved by the Ethics Committee in Research of the School of Dentistry, Federal University of Pelotas, under protocol number 55/2013. STROBE statement was used as a reporting guide.

In this cross-sectional retrospective study, the biopsies files from the Center of Diagnosis of Oral Diseases (CDOD) - School of Dentistry, Federal University of Pelotas - were retrieved, selecting those related to jaw disordes in young adults, aged 20 to 30 years, during the period comprised between January/1991 and December/2015. Oral soft tissue lesions observed in this age range and jaw lesions in individuals outside the above age range were excluded. Biopsy records that did not contain age information were not analyzed.

Data regarding sex, age, bone localization, clinical and histological diagnosis were collected. In relation to the anatomic site of the lesions, anterior and posterior regions were considered both for maxilla and mandible. Furthermore, bone localization could be classified into “more than one site” and “imprecise”.

The histological diagnosis for the biopsies were ranked under six categories, as it follows, partly according to the study of Lima *et al.* (2008) (13), which was conducted at the same Center: Cystic lesions of odontogenic origin, Periapical inflammation, Odontogenic tumors, Bone diseases, Health tissue and Nonspecific diagnostic. According to the 4th edition of the World Health Organization’s Classification of Head and Neck Tumors published in 2017, keratocystic odontogenic tumor was reclassified as cystic lesion of odontogenic origin (14).

Statistical analysis was performed using SPSS® version 22.0 for Windows (Chicago, IL, USA). The absolute and relative frequencies were estimated with descriptive analysis. Kappa statistic was performed to assess the concordance between clinical and histological diagnosis.

## Results

A total of 18,181 histopathological analysis was performed during the period of the study, being registered 1,599 jaw lesions in patients aged 20 to 30 years, which corresponds to 8.8% of the total cases. Only six cases of intraosseous lesions were considered inept, because their biopsy records had no information regarding age.

According to the age group studied (20 to 30 years), the average age was 24,59 years (SD 3,1). Nine hundred ninety-one (62%) lesions were found in females and 608 (38%) in males.

The distribution of lesions according their categories can be seen in  Table 1. More than half of pathologies were cystic lesions of odontogenic origin (51.4%), followed by periapical inflammation (17.6%). Regarding the site of the lesions, more than half (877/54.8%) occurred in posterior mandible, followed by posterior maxilla (339/21.2%), anterior maxila (305/19.1%) and anterior mandible (32/2%). Thirty six (2.3%) lesions extended over more than one site in maxilla or mandible. Ten (0.6%) jaw pathologies in the age range evaluated did not contain information about the site of ocurrence.

**Table 1:**
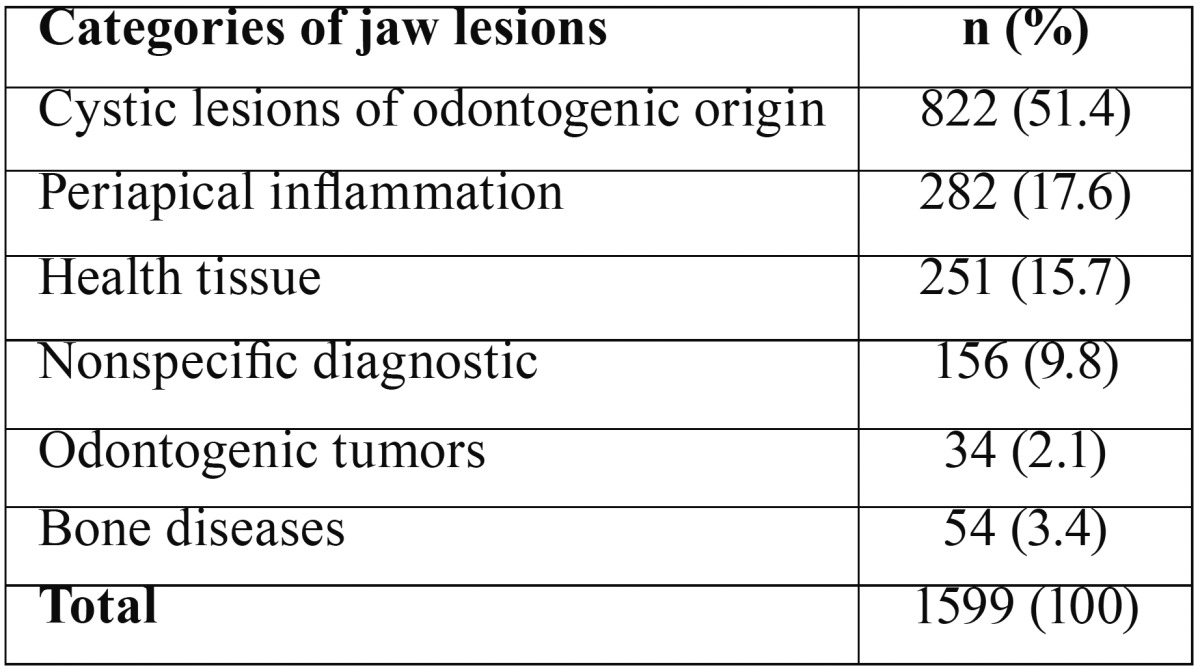
Absolute and relative frequencies of jaw lesions, according their categories (n=1599).

The seven most common histological diagnosis also present this pattern concerning the sites of occurrence and the ratio mandible/maxila is higher than one for most of these frequent pathologies ( Table 2). It is important to emphasize that these seven most frequent lesions correspond to a total of 1,429 evaluated cases (89.4%).

**Table 2:**
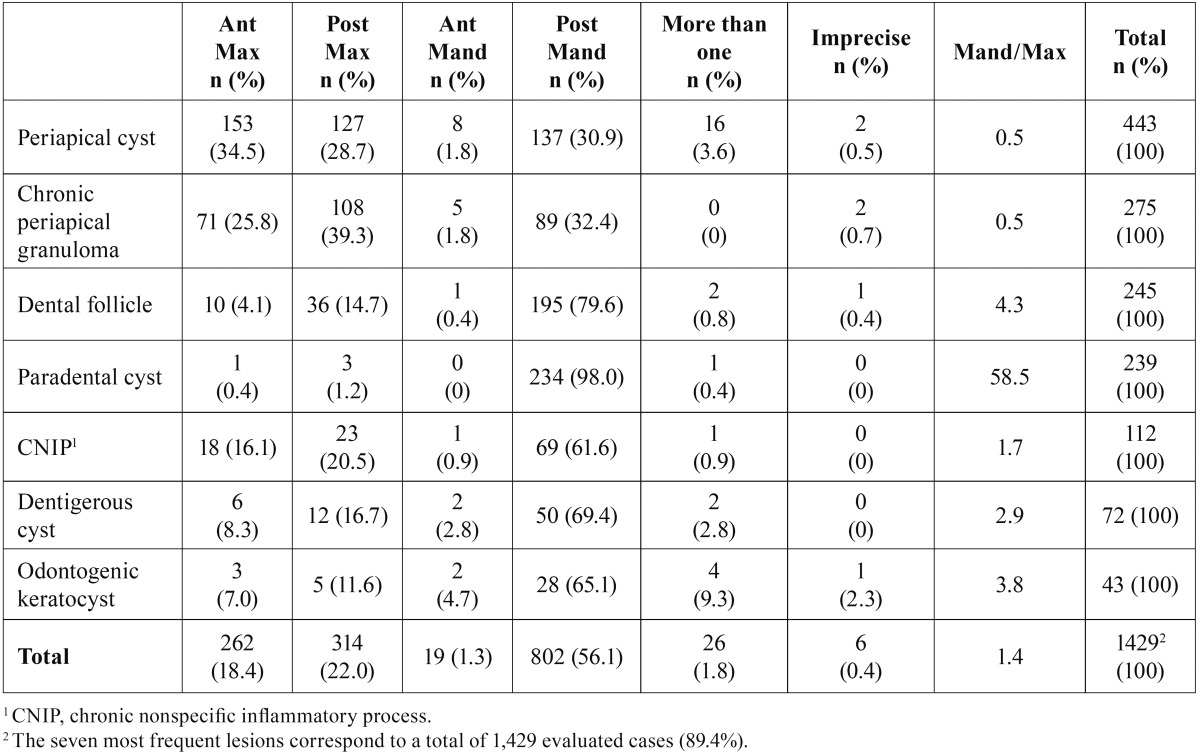
Most frequent histological diagnosis of jaw lesions and their sites.

Regarding to the analysis of concordance between the clinical diagnostic hypothesis established by the professional who performed the biopsy and the final histological diagnosis, it is important to highlight that in 196 (12.3%) cases, there was no information about the clinical diagnosis in biopsy records. The data can be seen in  Table 3. The general Kappa index was 0.5, which is considered moderate.

**Table 3:**
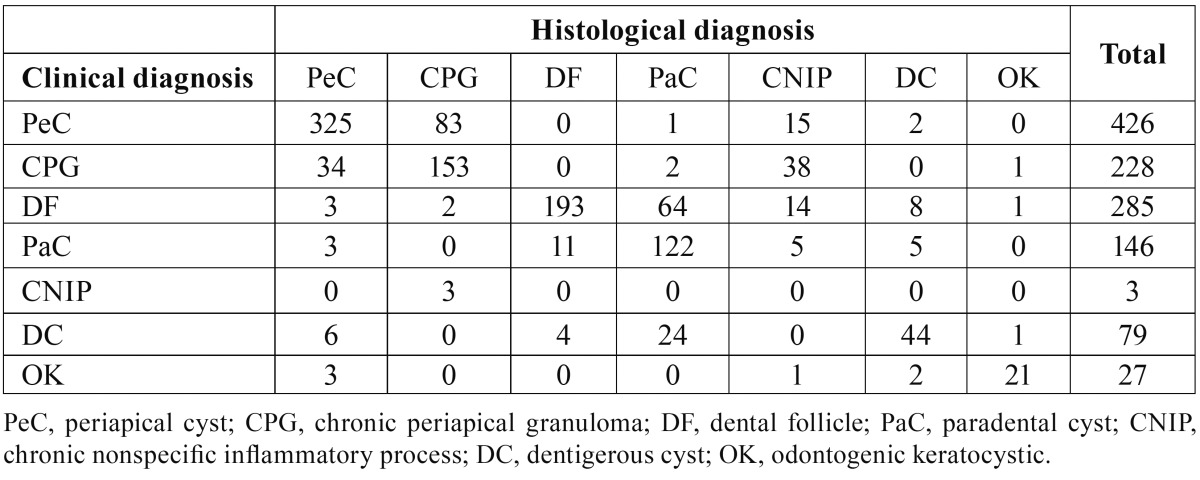
Clinical and histological concordance between the seven most frequent diagnosis of jaw lesions (n=1194).

## Discussion

The studies about the prevalence of oral lesions and their characteristics in specific populations are very important because they can provide relevant data related to an analyzed group of individuals, which can also facilitate their diagnosis and improve the knowledge about them. Similarly, long-term retrospective studies performed in services specialized in oral diagnosis can allow the establishment of a profile for these disorders (4,8,15), even though service-based studies are not designed to extrapolate results to the general population analysis.

The CDOD is a reference service, which receives biopsies from all the southern cities of the Rio Grande do Sul, a State located in extreme south of Brazil. This Center is an important reference in oral diagnosis for the topographic region in which it is located, accounting for more than 18,000 histopathological diagnosis in the last twenty-five years.

Yakin *et al.* (15) evaluated 616 intraosseous pathologic conditions from oral and maxillofacial regions, retrieved from a histopathological center of diagnosis. They found that more than 50% of the lesions occurred in individuals from second and third decades of life, varying from 1 to 90 years-old (15). In young adult population, it has been shown that not only the oral mucosal lesions, but also some intraosseous jaw disorders are very frequent (8,15). In relation to the sex of the patients, some variations in the frequency of the lesions are admitted, depending on the analyzed pathology. The present data shows a high prevalence of gnatic intarosseous lesions in females (62%).

Odontogenic cystic lesions were seen in more than half of the total cases (822/51.4%), being inflammatory or developmental, according to their origin. This group comprises entities such as periapical cyst, paradental cyst, dentigerous cyst and odontogenic keratocyst, which are all common lesions considering jaw pathologies (16-18). The periapical cyst was the most common inflammatory lesion and the dentigerous cyst the most prevalent developmental cyst, just as found in the literature (15,18).

The second more frequent group was the one represented by the periapical inflammations (282 cases/7.6%), mainly composed by the periapical granulomas, that together with the periapical cysts account to 718 (44.9%) of the studied lesions. Our findings match the literature data, which have pointed out high prevalence of these oral pathological entities, even when soft tissue lesions are also evaluated (16,18). Both periapical granulomas and periapical cysts are caused by pulpal injury due to caries or trauma (19).

Considering the dental follicle (15.3%), the dentigerous cyst (4.5%), and the paradental cyst (19%), the difficulties in the establishment of the differential diagnosis among them are well known (20). The literature has reported that radiolucencies involving the dental crown and measuring up to 3 mm could represent a normal dental follicle (20,21). On the other hand, lesions with the same radiographic aspect measuring above 3 mm could be considered pathological ones, such as dentigerous cysts or paradental cysts (20,21). Nonetheless, in a practical point of view, the association among the clinical, histopathological and imaginological characteristics is fundamental for the definitive diagnosis and the measurement standard is not enough for determining it (20-22).

Odontogenic tumors were responsible for 34 (2.1%) cases, being 11 (0.7%) multicystic ameloblastoma, 12 (0.7%) odontomas, 6 (0.4%) unicystic ameloblastomas, 3 (0.2%) odontogenic myxomas, and 2 (0.1%) cementoblastomas. No cases of other variants of ameloblastoma were found. It is important to highlight that in agreement with the new classification of Head and Neck Tumors ameloblastoma multicystic type refers to the conventional solid/multicystic (14). Also, none malignant odontogenic tumors were found in the studied group. The findings corroborate the literature, in which odontoma and ameloblastoma are reported as the most frequent odontogenic tumors in young adults (8,22). Sevrato *et al.* (8) reported data retrieved from another brazilian oral pathology service, in a period of 31 years and demonstrated that 40% of the odontogenic tumors occured between 20-29 years-old, being the majority benign.

Bone diseases account to 54 (3.4%), including mainly benign conditions such as fibrosseous lesions, osteomas, giant cell central granulomas and simple osseous cyst. Rare malignant lesions such as peripheral nerve sheath tumor, and osteossarcoma were also observed. Although the bone diseases group presents low prevalence, it comprises lesions with enough relevant clinical meaning in terms of their management and follow-up, being important to mention them (24,25).

One of the seven more prevalent conditions was chronic nonspecific inflammatory process (CNIP). As the own title reveals, there wasn’t a way to present a precise diagnosis in such cases, due to the absence of some clinical information and/or radiographic examination. Besides that, in some cases, the histopathological features were not too specific, leading to a more general diagnosis. CNIP was included in the group of nonspecific diagnosis. This last one accounts to 156 cases (9.8%). Therefore, it is fundamental to reinforce the need for complete documentation and information that may be sent along with the biopsies (20). Moreover, the difficulties during the surgical procedures must be registered, like the report of an incomplete excision or unsatisfactory material collection in cases of incisional biopsies. These records will help and guide the pathologist to establish the definitive diagnosis.

Regarding this, another important factor to be considered is the lack of data about the clinical diagnosis in the biopsy files, observed in 196 (12.3%) cases. This information is very important, because it can guide the pathologist, helping him to establish the final histopathological diagnosis (6,26).

Additionally to the missing data, another limitation of our study was that we could not analyze the radiographies of the majority of the cases, since expressive part of the specimens histologically examined did not have attached images. It needs to be strongly encouraged that the surgeons do it, due to the great importance of radiographic images in the diagnosis of intraosseous lesions.

Regarding the sites of occurence, more than 50% of the cases were located in posterior mandible, the same as stated in the literature (8,16,17). Posterior maxilla was the second site more prevalent, like observed in other studies (3,17). Still in relation to location it was observed that, among the seven more frequent lesions, the periapical cyst was more common in anterior maxilla, which can be explained by the high frequency of traumas in this region (17). Periapical granuloma was more commonly observed in posterior maxilla and its presence can be justified by the strong correlation with caries, frequently observed in posterior teeth (1,18). Dental follicle and paradental cyst occurred mainly in posterior mandible, due to the high frequency of impacted teeth in this particular region, especially third molars (17,20). The studied age group still commonly has those teeth in mouth, and they are usually in partial eruption, favoring the development of some lesions (27). Similarly, dentigerous cyst and odontogenic keratocyst were found preferably in posterior mandible, also corroborated by the literature (8,16,17).

General kappa index for the concordance between the clinical and histological diagnosis was 0.5, which is considered a moderate one. The biggest disagreement was between periapical cyst and periapical granuloma, already expected, since such injuries can only be differentiated through the microscopic analysis (26). In the same way the concordance between dental follicle and para-dental cyst was low. Also between dentigerous cyst and paradental cyst. These facts could be explained by the clinical and imaging similarities among them (20,21,26). Therefore, the possibility of a lesion being inflammatory or developmental condition must be lifted when it is removed from an impacted third molar (20). Regarding odontogenic keratocyst the concordance between clinical and histopathological diagnosis was satisfactory, a positive fact, considering the peculiar characteristics of this pathology, which reflect in its treatment and prognosis (23).

It is important to point out that our results are from a reference center and based on a histological analysis, therefore the prevalence of jaw injuries diagnosed in young adults aged 20 to 30 years can be higher than the one presented in this study.

Finally, the findings confirm the data from the literature about the most frequent jaw pathologies in young adults. They serve as subsidy for preventive measures of some entities, and are important in the improvement of the formulation of differential diagnosis and in the patient management.
